# Oral mucosal lesions in the mouth as first sign of dermatological diseases and disorders

**DOI:** 10.3205/dgkh000499

**Published:** 2024-10-02

**Authors:** Harepriya Meganathan Karthikeyini, Karthik Shunmugavelu, Evangeline Cynthia Dhinakaran

**Affiliations:** 1 Davao Medical School Foundation, Davao City, Philippines; 2 Department of Dentistry, PSP Medical College Hospital and Research Institute Tambaram, Panruti, India; 3 Department of Pathology, Sree Balaji Medical College and Hospital, Chrompet, Chennai, Tamilnadu, India

**Keywords:** oral mucosal lesion, Wickham striae, Nikolsky sign, pemphigoid

## Abstract

Oral mucosal lesions manifest as a first sign of immune-mediated disorders. Lichen planus, pemphigus and pemphigoid are the most frequent immunologically mediated mucocutaneous diseases with oral involvement. Oral lesions are initially detected by dental health practitioners. Early detection can help in appropriate treatment and better quality of life.

Based on an analysis of 6,300 medical records from the period 1997 to 2018, 105 (1.66%) were attributable to these immunologically mediated diseases, of which 86 (1.36%) were due to lichen planus, 4 (0.06%) to pemphigus and 15 (0.23%) to pemphigoid.

## Abstract (de)

Läsionen der Mundschleimhaut sind ein erstes Anzeichen für immunvermittelte Erkrankungen. Lichen planus, Pemphigus und Pemphigoid sind die häufigsten immunologisch vermittelten mukokutanen Erkrankungen mit oraler Beteiligung. Häufig wird der Zahnarzt als erster mit diesen Läsionen konfrontiert. Daher ist ihre Identifizierung, die ausschließlich auf oralen Läsionen beruht, eine Herausforderung für Zahnärzte, um eine Verzögerung bei der Diagnosestellung und der geeigneten Behandlung des Patienten zu vermeiden.

Nach Auswertung von 6.300 Krankenakten aus dem Zeitraum 1997 bis 2018 entfielen 105 (1,66 %) auf diese immunologisch vermittelten Erkrankungen, davon 86 (1,36 %) auf Lichen planus, 4 (0,06 %) auf Pemphigus und 15 (0,23 %) auf Pemphigoid.

## Introduction

Lichen planus, pemphigus, and pemphigoid are the most frequent immunologically mediated mucocutaneous diseases with oral involvement. Frequently, the first manifestations of these systemic illnesses are plaques or vesiculobullous, ulcerative, and/or erosive oral lesions. It is important to mention that the lesions in these 3 diseases and others (infectious and non-infectious) have identical clinical and demographic characteristics. Hence, the identification of a disease based purely on oral lesions is a challenge for dentists, which leads to a delay in the confirmation of the correct diagnosis and ideal management of the patient. This is especially crucial considering that pemphigus is a life-threatening disease with poor prognosis, and an early diagnosis is critical for successful treatment [1], [2], [3], [4], [5], [6]. 

In lichen planus, T-lymphocytes are activated, leading to the destruction of the epithelial basal cell layer by apoptosis [7]. A possible connection between lichen planus and infectious diseases, such as chronic hepatitis C, has been revealed [8]. While still a matter of debate, lichen planus is considered at risk of malignant transformation, according to the latest World Health Organization classification of tumors [9], [10], [11], [12]. Pemphigus is a severe and life-threatening autoimmune chronic mucocutaneous disorder. In this condition, mainly IgG serum autoantibodies are raised against cadherin-type cell adhesion molecules of the squamous epithelium, generally desmoglein [4].

Oral lesions are a hallmark of pemphigus and often herald the disease, being detected in almost every patient [13], [14], [15]. It is recognised that it has a reasonably strong genetic background, with a higher presence among people from the Mediterranean and South Asia and in certain ethnic groups, such as Ashkenazic Jews [16]. In pemphigoid, mostly the oral mucosa is the first affected site and it is solely involved in 85% of cases in the literature [5], [17], [18]. Further, a few patients may exhibit systemic and severe complications, e.g., ocular involvement, which may result in symblepharon (adhesion between the eyelid and the eyeball) and blindness [17], [18]. 

The purpose of this study was to use medical records to retrospectively analyze and compare the demographic data and clinical manifestations of oral pemphigus, oral lichen planus (OLP), and oral pemphigoid.. The authors aim to provide related information about the similarities and variances among these diseases, helping dentists to improve their ability to properly diagnose the main clinical presentations of each disease.

## Materials and methods

Biopsy charts were taken from the records of the archives section “Cases”, and those with a histopathological diagnosis of oral lichen planus, oral pemphigus and oral pemphigoid were chosen. HE-stained sections were re-examined according to the current criteria. 

Cases having clinical and histopathological features suggestive of lichenoid reaction were excluded. Demographic data – gender and age, along with the clinical features of the oral lesion (site, color, size, number of lesions, recurrence, evolution time, and symptoms) – were extracted from the charts. Symptoms in oral lesions were classified as pain or a burning/itching sensation. The ability to spell clinical presentations of oral lichen planus, oral pemphigus and oral pemphigoid was studied, comparing the clinical diagnostic hypotheses described in the charts with the final histopathological diagnosis. Explanatory statistical analysis was carried out using SPSS version 21.

## Results

Of all the 105 cases found with a history of immunological deficit diseases (1.66% of all studied records), oral lichen planus accounted for 86 (1.36%) of them, oral pemphigus 4 (0.06%), and oral pemphigoid 15 cases (0.23%). 

The prevalence of a positive Nikolsky’s sign (a “slipping away” of the skin; a common lesion in oral pemphigus) was noticed in all 4 (3.8%) cases.

Clinical and histopathological data are spelled in Table 1 (Tab. 1).

## Discussion

**Oral pemphigus**: Histopathological features of perilesional biopsy specimens of oral pemphigus displayed a characteristic intraepithelial suprabasilar clefting with acantholysis (Tzanck) cells, which tended to present a round shape. The basal layer cells remained adhered to the underlying basement membrane zone and a mild to moderate chronic inflammatory infiltrate was generally seen in the connective tissue. The patients’ ages ranged from 27 to 43 years, with a mean age of 31.5 years. Equal predilection was noticed in males and females with a ratio of 1:1. Out of the 4 cases (3.8%) diagnosed with oral pemphigus, two lesions were found mainly in the buccal mucosa and the other two lesions on the gingiva. The most commonly noticed clinical presentation was of multiple ill-defined erythematous (red, swollen) ulcers, erosions and burning sensation. Histopathological features contained suprabasilar split, acantholysis, Tzanck cells, and relative scarcity of intense inflammatory cell infiltrate. 

Immune-mediated mucocutaneous disease may display oral involvement in which a pathological process promotes the loss of epithelial integrity. The primary etiology of these conditions are not fully understood, although cellular and/or humoral immune responses are thought to play a major role. Such immune responses are directed against epithelial or connective tissue in a chronic and recurrent pattern [3], [6], [18], [19]. 

This study evaluated the 3 often immunologically mediated diseases with oral manifestations: oral lichen planus, oral pemphigus, and oral pemphigoid. Taken together, all three lesions accounted for 1.7% of all the studied records. In our study, the oral pemphigus female:male ratio was 1:1, which agrees with the literature regarding the gender predilection [5], [14]. Oral pemphigus usually presents as multiple symptomatic erythematous ulcers on the buccal mucosa of the 2 women in this study (27 and 43 years of age) and the gingiva of the 2 men included in this study (23 and 33 years of age). The careful evaluation of the lesion’s presentation is vital to achieve the correct clinical diagnosis. It also must be noted that the “classic” characteristics of each disease are not always observed in each patient; thus, differential diagnosis may be a clinical challenge.

Healthcare professionals should be well versed with these diseases and carry out a careful examination of the patients’ oral mucosa. The sites most affected by oral pemphigus were the buccal mucosa and gingiva, and ulcers were often seen in oral pemphigus. Multiple lesions occur in oral pemphigus patients, showing the importance of carefully screening the oral mucosa of these patients at every appointment, so that the best clinical assistance and treatment are provided. According to the outcome of this study, the size of the lesion does not appear to be important for diagnosis. The presence of a positive Nikolsky’s sign may be useful for diagnosis. 

The term “pemphigus” derives from Greek “pemphix” (bubbles, blisters). Pemphigus vulgaris (PV) was the name given to this diesease by Wickman in 1791. PV is a frequently observed member of a group of chronic autoimmune mucocutaneous diseases characterized by the development of intraepithelial blisters. Pemphigus is divided into pemphigus vulgaris, pemphigus erythematosus vegetans type, pemphigus foliaceus, paraneoplastic pemphigus, IgA type [16], [17], [18], [19], [20], [21], [22], [23], [24], [25], [26], [27], [28], [29], [30], [31], idiopathic type, followed by staphylococcal scalded-skin syndrome. In pemphigus, the antibodies are produced against desmosomes (adhesive protein); the main antigen in oral pemphigus is desmoglein (Dsg)3, a protein constituent of the desmosomes. Most patients with PV have circulating IgG auto antibodies against Dssg3. These antibodies bind to the Dsg3on the epithelial cell membrane and may evoke acantholysis. Another desmoglein is Dsg, which is the target of auto-antibody formation in pemphigus foliaceus. Patients with oral lesions can be initially misdiagnosed, usually as aphthous stomatitis, gingiyostomatitis, erythema multiforme, erosive lichen planus or oral candidiasis, and may thus receive the wrong treatment for months or years [32], [33], [34], [35].

Globally, there are 0.5 to 3.2 cases reported every year per 100,000 population with the highest incidence in the 5th and 6th decade of life, and male:female ratio of 1:2. Some rare cases have been reported in children and the elderly. Oral pemphigus is the most common variant of potentially fatal autoimmune diseases characterized by cutaneous or mucosal blistering, and shows oral lesions as early manifestations of the disease in nearly 50% of the cases [20], [21]. Its peak incidence is between the fourth and fifth decade of life [22]. Clinically, oral lesions precede skin lesions in most cases and show as blisters which rupture, quickly resulting in painful erosions. Buccal mucosa, lips, and soft palate are generally involved [23]. Diagnosis is done on the basis of identification of clinical manifestations and validation through biopsy. Demonstration of immunoglobulins in the spinous-cell junctions by distinct immune fluorescence (IF) is often used for the final confirmation [24], [25]. As the oral appearance of the disease is often the first indicator that can lead to the final diagnosis, it is very important for the dental practitioner to spot the oral lesions of oral pemphigus at a sufficiently early stage to begin further investigations and treatment.

Differential prognosis of oral pemphigus comprises:

- Recurrent aphthous stomatitis – ulcers (aphthae) in oral mucosa with yellowish base surrounded by an erythematous halo and regular margins, and which vanish without treatment
- Behçet’s illness – aphthae in the oral mucosa with genital and ocular ulcers
- Erythema multiforme – bull’s eye-shaped skin lesions, oral erosions, involvement of lips manifesting as erosions and crusts
- Erosive lichen planus – Wickham striae and erosive lesions
- Acute herpetic gingivostomatitis – prodromic symptoms followed by the onset of small yellowish vesicles that quickly rupture, resulting in ulcers with a reddened halo; affects free and attached gingivae.
- Impetigo – infection with presence of skin ulcers coated by a honey-colored crust. It affects face, arms and legs. It is more frequent in children.
- Disease due to linear IgA deposit – blisters and pruritic lesions, target-shaped lesions
- Mucosal pemphigoid or cicatricial pemphigoid – manifestation of an underlying malignant disease, dental lesions follow skin lesions, and blisters are smaller with a shorter duration than in PV, heal quickly without scarring
- Bullous pemphigus – tension blisters with lucid contents which show on normal or erythematous skin; severe pruritus, symmetric lesions that appear on flexion spots, root of extremities, thighs and abdomen; scarce on mucosa
- Herpetiform dermatitis – 1–3 cm erythemas that penetrate palate and buccal mucosa; aphthae on labial mucosa; present months or years after the appearance of skin lesions.
- Epidermolysis bullosa – blisters with minimal pressure, ring-shaped atrophic scars on the inner surface of limbs and joints
- Paraneoplastic pemphigus – syndrome connected to lymphoproliferative neoplasm of B cells
- No oral lesions seen in erythematous type and pemphigus foliaceus
- Chronic benign pemphigus familiaris – generally no oral lesions
- Disseminated lupus erythematosus – fever, asthenia usually accompanied by petechiae edemas and dry mouth
- Crohn’s disease and hemorrhagic rectal colitis – symptoms accompanied by abdominal pain, aphthae in oral mucosa, asthenia, weight loss and anorexia.
- Folic acid or Vitamin B12 deficiency/deficit – dental pain, erythematous tongue, asthenia and anemia, parasthesias in limbs and physical problems, hypochromic iron deficiency pallor, tiredness, cephalalgias, vertigo, murmur in the ears, irritability, sleeplessness, focus problems, sensitivity to cold, anorexia and nausea.
- Enteropathic acrodermatitis – loss of taste and smell, vision problems, severe diarrhea, alopecia and hypertension [36], [37], [38], [39], [40], [41], [42], [43], [44].

## Conclusions

From the results of this study, it can be inferred that immune-mediated diseases with oral manifestations are comparatively scarce among oral lesions. The present study comprises the retrospective assessment of oral immunologically mediated diseases, in which data regarding the 3 diseases were extracted and spelled altogether. As these diseases can often present similar clinical features and demographic data, their exact recognition can be a challenge for dentists. 

A perfect and early diagnosis is of significant importance for a proper therapeutic decision and correct approach to the patient’s treatment. For this purpose, despite the advent of new, promising drugs in the market, immunosuppressive (mainly corticosteroids) are still the mainstay of treatment, and their harmful side effects must be seriously weighed against the benefits.

Considering the relatively low prevalence of oral manifestations of immune-mediated diseases, large epidemiological studies regarding these conditions are clearly required. Moreover, to fill in patients’ charts properly, it is essential to control research bias related to missing data and to provide more reliable information on this issue.

## Notes

### Competing interests

The authors declare that they have no competing interests.

### Authors’ ORCID

- Karthikeyini HM: 0009-0003-7788- 9872
- Shunmgavelu K: 0000-0001-7562-8802
- Dhinakaran EC: 0000-0003-2194-6455

## Figures and Tables

**Table 1:**
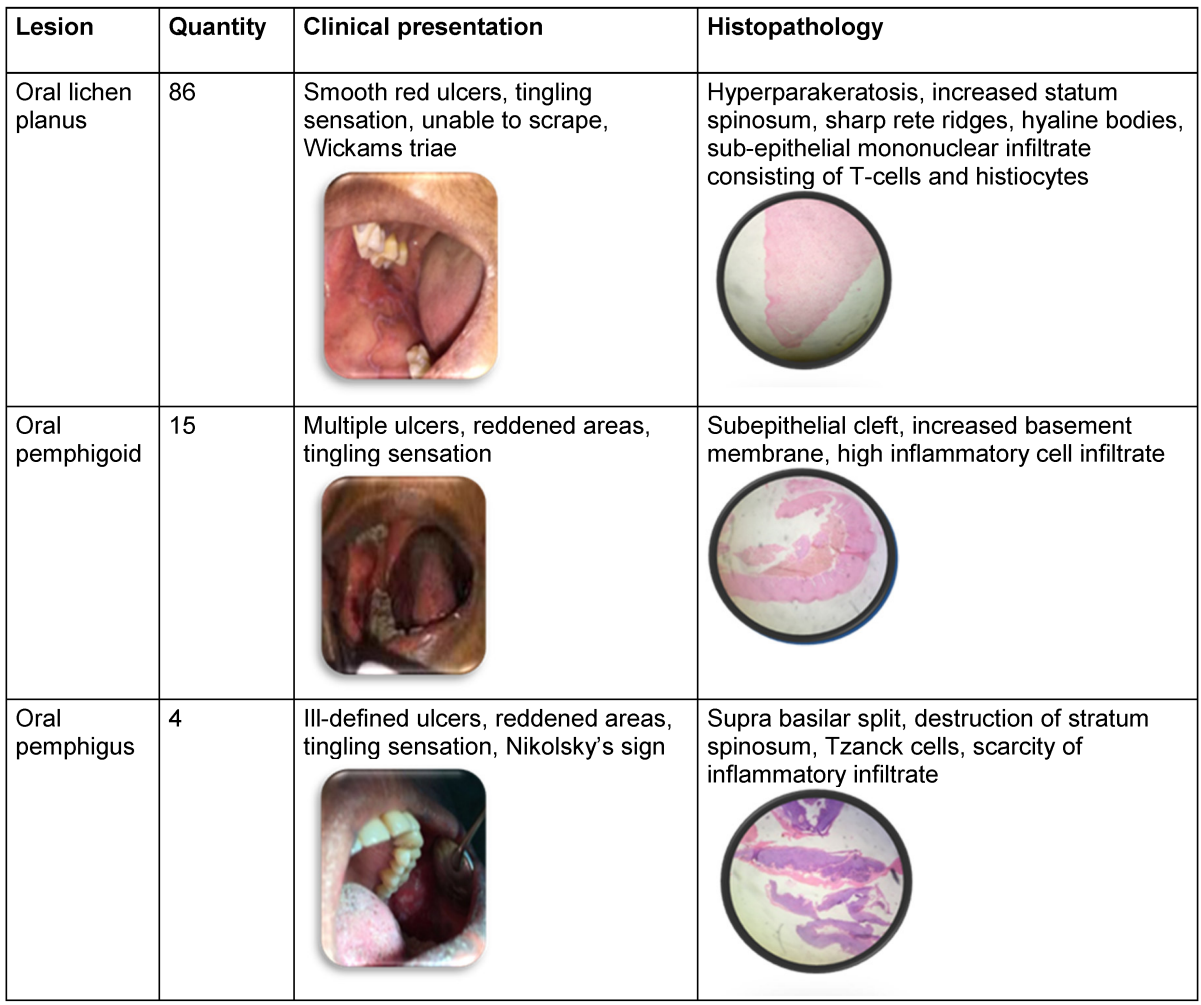
Clinical and histopathological features of oral mucosal lesions
